# Maternal Exposure to Ambient Levels of Benzene and Neural Tube Defects among Offspring: Texas, 1999–2004

**DOI:** 10.1289/ehp.1002212

**Published:** 2010-10-05

**Authors:** Philip J. Lupo, Elaine Symanski, D. Kim Waller, Wenyaw Chan, Peter H. Langlois, Mark A. Canfield, Laura E. Mitchell

**Affiliations:** 1 Division of Epidemiology, Human Genetics and Environmental Sciences, University of Texas School of Public Health, Houston, Texas, USA; 2 Human Genetics Center and; 3 Division of Biostatistics, University of Texas School of Public Health, Houston, Texas, USA; 4 Birth Defects Epidemiology and Surveillance Branch, Texas Department of State Health Services, Austin, Texas, USA

**Keywords:** air pollution, benzene, birth defects, BTEX, epidemiology, hazardous air pollutants, maternal exposure, neural tube defects

## Abstract

**Background:**

Previous studies have reported positive associations between maternal exposure to air pollutants and several adverse birth outcomes. However, there have been no studies assessing the association between environmental levels of hazardous air pollutants, such as benzene, and neural tube defects (NTDs), a common and serious group of congenital malformations.

**Objective:**

Our goal was to conduct a case–control study assessing the association between ambient air levels of benzene, toluene, ethylbenzene, and xylene (BTEX) and the prevalence of NTDs among offspring.

**Methods:**

The Texas Birth Defects Registry provided data on NTD cases (spina bifida and anencephaly) delivered between 1999 and 2004. The control group was a random sample of unaffected live births, frequency matched to cases on year of birth. Census tract–level estimates of annual BTEX levels were obtained from the U.S. Environmental Protection Agency 1999 Assessment System for Population Exposure Nationwide. Restricted cubic splines were used in mixed-effects logistic regression models to determine associations between each pollutant and NTD phenotype.

**Results:**

Mothers living in census tracts with the highest benzene levels were more likely to have offspring with spina bifida than were women living in census tracts with the lowest levels (odds ratio = 2.30; 95% confidence interval, 1.22–4.33). No significant associations were observed between anencephaly and benzene or between any of the NTD phenotypes and toluene, ethylbenzene, or xylene.

**Conclusion:**

In the first study to assess the relationship between environmental levels of BTEX and NTDs, we found an association between benzene and spina bifida. Our results contribute to the growing body of evidence regarding air pollutant exposure and adverse birth outcomes.

Birth defects are the leading cause of infant mortality in the United States (Petrini et al. 2002), and more than 65% are of unknown origin (Bale et al. 2003). Neural tube defects (NTDs), one of the most common groups of birth defects, are complex malformations of the central nervous system that result from failure of neural tube closure (Christianson et al. 2006). Infants with NTDs experience both increased morbidity and mortality compared with their unaffected contemporaries (Mitchell et al. 2004; Wong and Paulozzi 2001). Although these defects are clinically significant, little is known about their etiology.

Hazardous air pollutants (HAPs), toxic substances commonly found in the air environment, are known or suspected to cause serious health effects [U.S. Environmental Protection Agency (EPA) 2007a]. HAPs are a heterogeneous group of pollutants that include organic solvents such as benzene, toluene, ethylbenzene, and xylene (BTEX) and are emitted from several sources. Human exposure to HAPs can result from inhalation, ingestion, and dermal absorption. Benzene is one of the most prevalent HAPs in urban areas (Mohamed et al. 2002) and is of particular interest because it has been associated with several adverse health outcomes including pediatric cancer and intrauterine growth restriction [International Agency for Research on Cancer (IARC) 1982, 1987; Slama et al. 2009; U.S. EPA 2007a; Whitworth et al. 2008; Yin et al. 1996].

Some studies have reported positive associations between maternal exposures to air pollutants other than HAPs (i.e., criteria pollutants) and birth defects, including ozone and certain cardiac defects (Gilboa et al. 2005; Ritz et al. 2002), ozone and oral clefts (Hwang and Jaakkola 2008), and particulate matter (PM) and nervous system defects (Rankin et al. 2009). Other studies have been inconclusive regarding the role of criteria pollutants on the prevalence of oral clefts (Hansen et al. 2009; Marshall et al. 2010) and congenital heart defects (Hansen et al. 2009; Strickland et al. 2009).

Occupational studies have demonstrated a positive association between maternal exposure to organic solvents (e.g., benzene) and birth defects, including NTDs (Brender et al. 2002; McMartin et al. 1998; Wennborg et al. 2005). Despite this association, no studies have assessed the effect of environmental levels of benzene or other HAPs on NTD prevalence. Therefore, we conducted a study to assess the association between maternal exposure to environmental levels of BTEX and the prevalence of NTDs in offspring. Benzene was the primary pollutant of interest because of its association with other adverse outcomes (IARC 1982; Whitworth et al. 2008). Toluene, ethylbenzene, and xylene were selected for investigation because of their association with benzene (Mohamed et al. 2002). This study was conducted in Texas, a state that ranks number one in the United States for benzene levels in ambient air and accounts for 48% of all benzene emissions in the nation (U.S. EPA 2007b).

## Materials and Methods

### Study population

Data on live births, stillbirths, and electively terminated fetuses with NTDs (spina bifida and anencephaly) delivered between 1 January 1999 and 31 December 2004 were obtained from the Texas Birth Defects Registry (*n* = 1,108) (Texas Department of State Health Services 2010). The registry is a population-based, active surveillance system that has monitored births, fetal deaths, and terminations throughout the state since 1999. We selected a stratified random sample of unaffected live births delivered in Texas between 1 January 1999 and 31 December 2004 as the control group, using a ratio of four controls to one case. Controls were frequency matched to cases by year of birth because of the decreasing birth prevalence of NTDs over time (Canfield et al. 2009a). This yielded a group of 4,132 controls. The study protocol was reviewed and approved by the institutional review boards of the Texas Department of State Health Services and the University of Texas Health Science Center at Houston.

### Exposure assessment

Census tract–level estimates of ambient BTEX levels were obtained from the U.S. EPA 1999 Assessment System for Population Exposure Nationwide (ASPEN) (Rosenbaum et al. 1999; U.S. EPA 2006, 2008). The methods used for ASPEN have been described fully elsewhere (Rosenbaum et al. 1999; U.S. EPA 2006). Briefly, ASPEN is part of the National Air Toxic Assessment (Ozkaynak et al. 2008) and is based on the U.S. EPA Industrial Source Complex Long-Term Model. It takes into account emissions data, rate, location, and height of pollutant release; meteorological conditions; and the reactive decay, deposition, and transformation of pollutants. Ambient air levels of BTEX are reported as annual concentrations in micrograms per cubic meter (U.S. EPA 2006). Residential air levels of BTEX were estimated based on maternal address at delivery as reported on vital records for cases and controls. Addresses were geocoded and mapped to their respective census tracts by the Texas Department of State Health Services.

### Potential confounders

Information on the following potential confounders was obtained or calculated from vital records data: sex of infant; year of birth; maternal race/ethnicity (non-Hispanic white, non-Hispanic black, Hispanic, or other); maternal birth place (United States, Mexico, or other); maternal age (< 20, 20–24, 25–29, 30–34, 35–39, or ≥ 40 years); maternal education (less than high school, high school, or more than high school); marital status (married or not married); parity (0, 1, 2, or ≥ 3); maternal smoking (no or yes); and season of conception (spring, summer, fall, or winter). Additionally, as the exposure assessment for BTEX was based on census tract–level estimates, we opted to include a census tract–level estimate of socioeconomic status (percentage below poverty level), which was obtained from the U.S. Census 2000 Summary File 3 (U.S. Census Bureau 2010). Percentage of census tract below poverty level was categorized into quartiles (low, medium-low, medium-high, and high poverty level) on the basis of the distribution among the controls.

### Statistical analysis

Frequency distributions for categorical variables were determined for controls and the two NTD subgroups (spina bifida and anencephaly). Correlations between levels of BTEX were determined using Spearman’s rank correlation. We used mixed-effects logistic regression to assess associations between each HAP and NTD phenotype while accounting for the potential within-group correlation resulting from the use of a census tract–level exposure assignment (Szklo and Nieto 2007). There is strong evidence that risk factor profiles are different for spina bifida and anencephaly (Canfield et al. 2009b; Khoury et al. 1982; Lupo et al. 2010b; Mitchell 2005); therefore, analyses were conducted separately in these phenotypes.

Based on plots assessing the trend between benzene levels and NTD prevalence, the exposure–outcome relationship appeared nonlinear; therefore we opted to use restricted cubic splines. Specifically, restricted cubic splines were fit to logistic regression models assessing the association between each HAP and NTD phenotype. The output from these models indicated four knots (corresponding to specific ambient HAP levels) where the exposure–outcome relationship changed. These knots were then used to determine cut points for low (i.e., reference), low-medium, medium, medium-high, and high ambient air levels (Durrleman and Simon 1989) and used in the final models assessing the association between each HAP and NTD phenotype. Because the low (i.e., reference) exposure category represents approximately 5% of the total population, we also defined the reference group as the 10th, 15th, and 20th percentile of exposure for each HAP, based on the distribution among controls, to assess how sensitive the results were to the cut point chosen for the reference group.

Variables were incorporated as confounders in the final models if inclusion resulted in ≥ 10% change in the estimate of effect between the air pollutant and NTD phenotype. Year of birth was included in each multivariable model, because it was a matching factor between cases and controls (Szklo and Nieto 2007). Associations between each HAP and NTD phenotype were considered significant when *p* < 0.05. To formally examine nonlinearity in the exposure–outcome relationship, a likelihood ratio test was used, comparing a full model (i.e., with both linear and cubic spline terms) to a reduced model (i.e., with a linear term only) at a significance level of *p* < 0.05 (Durrleman and Simon 1989). All analyses were conducted using Intercooled Stata, version 10.1 (StataCorp LP, College Station, TX) or SAS version 9.2 (SAS Institute Inc., Cary, NC).

## Results

To minimize etiologic heterogeneity within the case group, cases with an associated chromosomal abnormality or other syndrome (*n* = 75) and those with a closed NTD (i.e., lipomyelomeningocele, *n* = 88) were excluded. Additionally, cases with missing geocoded maternal address were excluded (*n* = 109). After these exclusions, 533 spina bifida and 303 anencephaly cases were available for analysis. Of the 4,132 controls, 437 were excluded because of missing geocoded maternal address. The final control group consisted of 3,695 unaffected births for analysis. The proportion of case and control mothers missing address information was similar (11.5% and 10.5%, respectively), and differences between those with and without maternal address at delivery were minor (≤ 5%) on demographic factors (results not shown). Compared with controls, case mothers were more likely to be Hispanic, born in Mexico, young, and less educated (Table 1).

**Figure 1:**
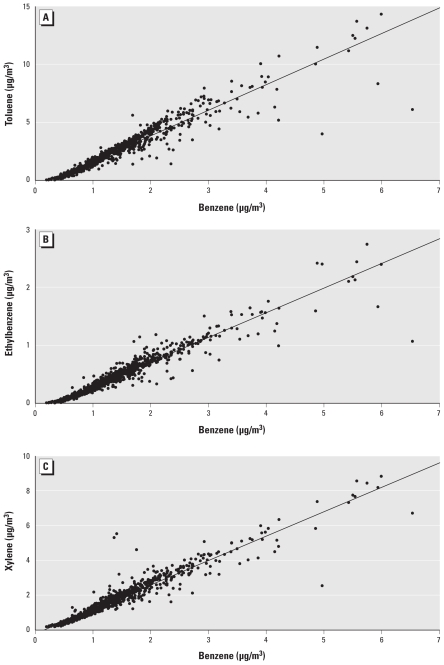
Scatterplots of (*A*) toluene and benzene, (*B*) ethylbenzene and benzene, and (*C*) xylene and benzene from the 1999 U.S. EPA ASPEN model for Texas census tracts included in the current analysis (*n* = 2,485).

Scatterplots of benzene and each of the other HAPs (toluene, ethylbenzene, and xylene) are presented in Figure 1. Levels of BTEX were highly and significantly correlated (ρ̂ ≥ 0.97, *p* < 0.001) (data not shown). Because of the high correlation between these compounds, statistical models including multiple pollutants were not assessed.

Results from the final models assessing the associations between BTEX and NTDs are presented in Table 2. After adjusting for year of birth, maternal race/ethnicity, education, census tract poverty level, and parity, mothers who lived in census tracts with the highest benzene levels were more likely to have offspring with spina bifida [odds ratio (OR) = 2.30; 95% confidence interval (CI), 1.22–4.33]. The degree of confounding from all covariates was modest; that is, adjusted ORs differed from crude ORs by no more than 15%. There were also positive associations with the low-medium (OR = 1.77; 95% CI, 1.04–3.00), medium (OR = 1.90; 95% CI, 1.11–3.24), and medium-high benzene exposure groups (OR = 1.40; 95% CI, 0.82–2.38). When the reference group was defined as less than or equal to the 10th, 15th, or 20th percentile of exposure, the association between maternal residence in a census tract with the highest benzene levels relative to the referent group and the prevalence of spina bifida remained, although it was attenuated (OR_10th_ = 1.96; 95% CI, 1.17–3.28; OR_15th_ = 1.59; 95% CI, 1.00–2.54; and OR_20th_ = 1.57; 95% CI, 1.00–2.46).

**Figure 2:**
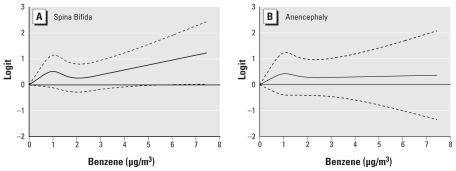
Restricted cubic splines representing the relationship between (*A*) benzene and the odds of spina bifida and (*B*) benzene and the odds of anencephaly. (Reference group is the lowest benzene exposure level; dashed lines represent 95% CIs.)

Based on the likelihood ratio test between the adjusted model with cubic splines and the model without the spline terms, there was a significant nonlinear relationship between maternal benzene exposure and spina bifida prevalence (*p* = 0.03). To further illustrate the nonlinear trend between benzene and NTDs, the estimated logits (and 95% confidence bands) were plotted against increasing benzene levels (Figure 2). For spina bifida, the logit appears to steadily increase when benzene levels are ≥ 3 μg/m^3^ and becomes statistically significant after benzene levels are approximately > 5 μg/m^3^ (Figure 2A), whereas no such trend was seen with anencephaly (Figure 2B).

## Discussion

We found a significant association between the prevalence of spina bifida in offspring and maternal exposure to ambient levels of benzene as estimated from the 1999 U.S. EPA ASPEN model (U.S. EPA 2006). The association was greatest for those in the highest exposure group. Positive associations between benzene and spina bifida were also observed in lower exposure categories; however, there was no monotonic dose–response relationship. Our finding that the risk of having a spina bifida-affected infant more than doubled for mothers living in census tracts with estimated benzene levels of ≥ 3 μg/m^3^ is in keeping with a report classifying individuals living in areas with benzene levels > 3.4 μg/m^3^ as being at the greatest risk for adverse health effects (Sexton et al. 2007). There were also associations with toluene, ethylbenzene, and xylene and between BTEX and anencephaly; however, these associations were not statistically significant.

The association between benzene levels and spina bifida appears to be nonlinear. This is supported by studies reporting nonlinear associations between personal exposure to benzene and various biomarkers (i.e., urinary metabolites and albumin adducts) of exposure using data collected on occupationally and environmentally exposed individuals, whereby exposure-metabolite curves became steeper at higher exposure levels (Kim et al. 2006; Lin et al. 2007).

Despite the strong correlations between the BTEX compounds, a significant association with spina bifida was seen only with benzene. Scatterplots of benzene and each of the other HAPs (toluene, ethylbenzene, and xylene) indicate that the correlations between pollutants are not as great at higher levels (Figure 1). In addition, we found lower correlations between benzene and the other pollutants (toluene, ethylbenzene, and xylene) when we restricted the analyses to census tracts with the highest benzene levels (*n* = 119) (ρ̂ = 0.62, 0.71, and 0.77, respectively).

Benzene is known to cross the placenta and has been found in cord blood at levels equal to or higher than maternal blood [Agency for Toxic Substances and Disease Registry (ATSDR) 2007]. Moreover, benzene can lead to genetic toxicity by covalently binding to DNA and forming DNA adducts, which, if not repaired, disrupt the microenvironment of the cell, leading to inhibition of important enzymes, cell death, and alteration of other cells (ATSDR 2007; Kim et al. 2006; Lan et al. 2004). If this occurs during the critical window of development, the complex cellular processes involved in neurulation (e.g., folate metabolism, cell proliferation, cellular adhesion, and vascular development) may be disturbed, resulting in NTDs.

Oxidative stress could also play a role in the teratogenic effect of benzene. Reactive oxygen species (ROS) formed after benzene exposure lead to DNA strand breakage and fragmentation leading to cell mutation (Hansen 2006; Xia et al. 2004). The importance of oxidative stress as a mechanism of teratogenesis is suggested by several animal studies (Fantel 1996). Treatment of pregnant rabbits and mice with ROS inhibitors diminished the effect of teratogens and reduced the amount of DNA oxidation (Liu and Wells 1995; Parman et al. 1999; Wells et al. 1997). One study conducted in rats demonstrated that increased embryonic oxidation resulted in failure of neural tube closure (Morriss and New 1979).

Positive associations between maternal occupational exposures to organic solvents and congenital malformations have been reported. One study assessing maternal occupational exposure to benzene reported an OR of 5.3 (95% CI, 1.4–21.1) for neural crest malformations (including NTDs) (Wennborg et al. 2005). In addition, among Mexican Americans, mothers occupationally exposed to solvents were 2.5 times as likely (95% CI, 1.3–4.7) to have NTD-affected pregnancies than control mothers (Brender et al. 2002). In a meta-analysis of five studies (not including the two previously discussed), mothers who were occupationally exposed to organic solvents had 1.6 times greater odds (95% CI, 1.2–2.3) of having an infant with a birth defect (including NTDs) (McMartin et al. 1998).

A potential limitation of this study is related to the exposure assessment, which relied on modeled predictions of ambient air levels of BTEX (i.e., the ASPEN model) and may have resulted in misclassification. Personal exposure is a function of outdoor and indoor pollutant levels, as well as individual behavior (i.e., time spent outdoors vs. indoors) (Lee et al. 2004). However, it has been shown that for benzene, the ASPEN model is a good surrogate for exposure measures based on personal monitoring (Payne-Sturges et al. 2004). The fact that ASPEN data were available only for 1999 and not for the entire study period is an additional potential limitation. This may be a suitable surrogate for other years, because the sources of HAPs (e.g., emissions from roadways and industrial facilities) were unlikely to change during the study period (Grant et al. 2007; Sexton et al. 2007; Whitworth et al. 2008). Additionally, information on maternal periconceptional use of folic acid and/or multivitamins (a potential confounder) was not available. However, this population represents pregnancies conceived after mandatory folic acid fortification (January 1998), and a recent study found little evidence of an association between NTDs and maternal folic acid intake or multivitamin use since fortification (Mosley et al. 2009). Finally, exposure misclassification due to use of maternal address at time of delivery is also a potential source of bias in this study. Because NTDs develop within the first 4 weeks after conception, address at delivery may be different than address during the critical window of exposure (Selevan et al. 2000). However, our own analyses using cases and controls from Texas included in the National Birth Defects Prevention Study, with complete residential information during pregnancy, suggest there was no significant change in benzene exposure assignment when using address at delivery versus address at conception (Lupo et al. 2010a).

Strengths of this study include the use of a population-based birth defects registry that employs an active surveillance system to ascertain cases throughout the state of Texas. This should limit the potential for selection bias. Furthermore, the Texas Birth Defects Registry includes information on pregnancy terminations, reducing any potential bias due to the exclusion of these cases. An additional strength was the use of a relatively small (census tract–level) measure of exposure. Using larger geographic units to estimate exposure (e.g., counties) may not capture the spatial variability of benzene (Pratt et al. 2004). Furthermore, separate analyses were conducted for spina bifida and anencephaly, as opposed to combining the groups into a single phenotype. This is important, as the effects of some exposures appear to be heterogeneous across the subtypes of NTDs (Lupo et al. 2010b; Mitchell 2005).

## Conclusions

This study provides the first assessment of the relationship between maternal exposure to ambient levels of BTEX and the prevalence of NTDs in offspring. Our analyses suggest that maternal exposure to ambient levels of benzene is associated with the prevalence of spina bifida among offspring. We believe that future investigations of air pollutants and NTDs should include additional measures of exposure (e.g., air pollutant monitoring and biomarker data) and additional covariate information (e.g., genotypes and nutrient status).

## Figures and Tables

**Table 1:** Characteristics of controls and NTD cases (spina bifida and anencephaly) in Texas, 1999–2004 [*n* (%)].

Characteristic	Controls (*n* = 3,695)	Spina bifida (*n* = 533)	Anencephaly (*n* = 303)
Sex of infant			

Female	1,828 (49.5)	251 (47.3)	165 (54.8)
Male	1,867 (50.5)	280 (52.7)	136 (45.2)

Maternal race/ethnicity			

Non-Hispanic white	1,344 (36.5)	191 (36.0)	89 (29.5)
Non-Hispanic black	430 (11.7)	54 (10.2)	30 (10.0)
Hispanic	1,773 (48.1)	280 (52.8)	176 (58.5)
Other	138 (3.7)	5 (0.9)	6 (2.0)

Maternal birthplace			

United States	2,592 (70.4)	355 (67.4)	180 (62.5)
Mexico	785 (21.3)	145 (27.5)	93 (32.3)
Other	306 (8.3)	27 (5.1)	15 (5.2)

Maternal age (years)			

< 20	501 (13.6)	76 (14.3)	57 (18.8)
20–24	1,099 (29.7)	158 (29.6)	93 (30.7)
25–29	966 (26.1)	141 (26.5)	78 (25.7)
30–34	754 (20.4)	119 (22.3)	58 (19.1)
35–39	323 (8.7)	31 (5.8)	13 (4.3)
≤ 40	52 (1.4)	8 (1.5)	4 (1.3)

Maternal education			

< High school	1,155 (31.7)	188 (36.4)	107 (37.4)
High school	1,195 (32.8)	169 (32.7)	94 (32.9)
> High school	1,292 (35.5)	160 (30.9)	85 (29.7)

Marital status			

Married	2,498 (67.7)	355 (67.1)	194 (64.2)
Not married	1,192 (32.3)	174 (32.9)	108 (35.8)

Parity			

0	1,314 (36.9)	190 (37.7)	93 (31.9)
1	1,170 (32.9)	157 (31.2)	82 (28.1)
2	679 (19.1)	95 (18.8)	63 (21.6)
≥ 3	396 (11.1)	62 (12.3)	54 (18.5)

Maternal smoking			

No	3,447 (93.9)	505 (95.5)	282 (95.3)
Yes	225 (6.1)	24 (4.5)	14 (4.7)

Census tract poverty level^a^			

Low	922 (25.0)	100 (18.8)	56 (18.5)
Medium-low	925 (25.0)	144 (27.0)	82 (27.1)
Medium-high	926 (25.0)	137 (25.7)	81 (26.7)
High	922 (25.0)	152 (28.5)	84 (27.7)

Season of conception			

Spring	807 (24.0)	106 (22.5)	59 (24.0)
Summer	798 (23.7)	127 (27.0)	56 (22.8)
Fall	876 (26.0)	122 (25.9)	72 (29.2)
Winter	887 (26.3)	116 (24.6)	59 (24.0)

a Based on percentage of census tract below the poverty level.

**Table 2:** Adjusted ORs (95% CIs) for the associations between 1999 U.S. EPA ASPEN modeled estimates of BTEX and NTDs in Texas, 1999–2004.

	Spina bifida			Anencephaly		
Pollutant	Pollutant level [μg/m^3^ (range)]	Cases/controls (*n*)	Adjusted OR^a^,^b^ (95% CI)	Pollutant level [μg/m^3^ (range)]	Cases/controls (*n*)	Adjusted OR^b^,^c^ (95% CI)
Benzene						

Low (reference)	0.12–0.45	19/195	1.00	0.12–0.44	13/186	1.00
Medium-low	> 0.45–0.98	174/1,093	1.77 (1.04–3.00)	> 0.44–0.98	92/1,106	1.36 (0.71–2.59)
Medium	> 0.98–1.52	167/1,100	1.90 (1.11–3.24)	> 0.98–1.52	98/1,103	1.49 (0.78–2.83)
Medium-high	> 1.52–2.86	138/1,130	1.40 (0.82–2.38)	> 1.52–2.81	86/1,115	1.24 (0.65–2.37)
High	> 2.86–7.44	35/177	2.30 (1.22–4.33)	> 2.81–7.44	14/185	1.28 (0.56–2.89)

Toluene						

Low (reference)	0.01–0.31	20/191	1.00	0.01–0.30	14/186	1.00
Medium-low	> 0.31–1.50	179/1,089	1.56 (0.95–2.58)	> 0.30–1.53	89/1,115	1.33 (0.70–2.54)
Medium	> 1.50–2.84	161/1,107	1.43 (0.87–2.37)	> 1.53–2.85	97/1,096	1.49 (0.78–2.84)
Medium-high	> 2.84–5.96	146/1,125	1.31 (0.79–2.18)	> 2.85–5.90	90/1,113	1.31 (0.69–2.51)
High	> 5.96–14.3	27/183	1.46 (0.78–2.75)	> 5.90–14.3	13/185	1.19 (0.52–2.72)

Ethylbenzene						

Low (reference)	0.01–0.04	21/190	1.00	0.01–0.04	15/183	1.00
Medium-low	> 0.05–0.25	178/1,089	1.46 (0.89–2.38)	> 0.04–0.25	91/1,109	1.23 (0.66–2.30)
Medium	> 0.26–0.51	161/1,110	1.36 (0.83–2.23)	> 0.25–0.51	98/1,103	1.34 (0.72–2.50)
Medium-high	> 0.52–1.10	140/1,130	1.18 (0.72–1.94)	> 0.51–1.08	88/1,112	1.17 (0.63–2.19)
High	> 1.11–2.74	33/176	1.72 (0.94–3.15)	> 1.08–2.74	11/188	0.90 (0.38–2.07)

Xylene						

Low (reference)	0.18–0.36	21/190	1.00	0.18–0.36	14/183	1.00
Medium-low	> 0.36–1.10	177/1,092	1.45 (0.88–2.36)	> 0.36–1.12	92/1,110	1.35 (0.70–2.58)
Medium	> 1.10–1.96	164/1,100	1.39 (0.85–2.27)	> 1.12–1.97	91/1,107	1.36 (0.71–2.60)
Medium-high	> 1.96–3.90	140/1,133	1.18 (0.72–1.94)	> 1.97–3.86	92/1,110	1.32 (0.69–2.52)
High	> 3.90–8.84	31/180	1.64 (0.90–3.01)	> 3.86–8.84	14/185	1.26 (0.56–2.85)

a Adjusted for year of birth, maternal race/ethnicity, and parity. (Model for benzene also included percentage of census tract below poverty level and maternal education.)

b Estimates from mixed-effects logistic regression models that account for group effects at the census tract level.

c Adjusted for year of birth, sex of infant, and season of conception.
